# Upregulation of VEGF and PEDF in Placentas of Women with Lower Extremity Venous Insufficiency during Pregnancy and Its Implication in Villous Calcification

**DOI:** 10.1155/2019/5320902

**Published:** 2019-12-09

**Authors:** Miguel A Ortega, Miguel Ángel Saez, Ángel Asúnsolo, Beatriz Romero, Coral Bravo, Santiago Coca, Felipe Sainz, Melchor Álvarez-Mon, Julia Buján, Natalio García-Honduvilla

**Affiliations:** ^1^Department of Medicine and Medical Specialties, Faculty of Medicine and Health Sciences and Networking Biomedical Research Centre on Bioengineering, Biomaterials and Nanomedicine (CIBER-BBN), University of Alcalá, Alcalá de Henares, Madrid, Spain; ^2^Ramón y Cajal Institute of Healthcare Research (IRYCIS), Madrid, Spain; ^3^Pathological Anatomy Service, Central University Hospital of Defence-UAH Madrid, Spain; ^4^Department of Surgery, Medical and Social Sciences, Faculty of Medicine and Health Sciences, University of Alcalá, Alcalá de Henares, Madrid, Spain; ^5^Service of Gynecology and Obstetrics, Section of Fetal Maternal Medicine, Central University Hospital of Defence-UAH Madrid, Madrid, Spain; ^6^Angiology and Vascular Surgery Service, Central University Hospital of Defence-UAH Madrid, Madrid, Spain; ^7^Immune System Diseases-Rheumatology and Oncology Service, University Hospital Príncipe de Asturias, Alcalá de Henares, Madrid, Spain; ^8^Internal Medicine Service, University Hospital Príncipe de Asturias, Alcalá de Henares, Madrid, Spain

## Abstract

Pregnancy is a period in a woman's life in which changes can occur that affect different physiological processes. Common conditions during this period include vascular changes, such as lower extremity venous insufficiency (VI). This is an observational, analytical, and prospective cohort study in which 114 pregnant women were analyzed, of which 62 were clinically diagnosed with VI. In parallel, 52 control patients without VI (HC) were studied. The aim of this study was to observe changes in angiogenesis and inflammation markers as well as the presence of calcium deposits. The expression of vascular endothelial growth factor (VEGF), transforming growth factor-*β* (TGF-*β*), and pigment epithelium-derived factor (PEDF) was analyzed by immunohistochemistry and RT-qPCR. The presence of calcium deposits was revealed using the von Kossa method. In the placentas of mothers with VI, gene expression of VEGF (34.575 [32.380–36.720] VI vs 32.965 [30.580–36.320] HC) and PEDF (25.417 [24.459–27.675] VI vs 24.400 [23.102–30.223] HC) significantly increased, as was protein expression in the placental villi. An increase in calcium deposits was observed in the placentas of women with VI (72.58% VI/53.84% HC). This study revealed the existence of cellular damage in the placental villi of mothers with VI with tissue implications such as increased calcification.

## 1. Introduction

The appearance of venous insufficiency (VI) in the lower extremities during pregnancy is a common complication, which is usually detected starting in the second half of pregnancy [1, 2]. VI is a vascular disorder that is defined by changes in the peripheral venous system and is a complication with a high prevalence in pregnancy [3, 4]. The incidence of this venous pathology increases with the number of pregnancies and fetuses and with family history [5]. In pregnant women, it has been found that venous compression by the fetoplacental organ is a very important inducing factor in the development of VI [6–8].

The repercussions of venous disorders in pregnant women are not fully known, but the different components of the placenta make it one of the most susceptible tissues to these repercussions. Our previous studies have demonstrated that VI is related to structural lesions of the placental villi associated with an increase in hypoxia-inducible factor (HIF) [7, 9]. The association of HIF with vascular diseases during pregnancy is well known; numerous studies have clarified its important role in the pathogenesis of diseases such as preeclampsia [10–12]. One of the key points in the regulation of these processes is the angiogenic inducer vascular endothelial growth factor (VEGF), which plays a role in alterations in placental pathology [13]. Recent studies have indicated a decrease in transforming growth factor-*β* (TGF-*β*) in the placenta of women with preeclampsia [14]. Notably, equilibrium between VEGF and pigment epithelium-derived factor (PEDF), both of which are associated with altered angiogenesis and vascular remodeling, is necessary [15, 16].

Cell damage can be associated with a process of connective tissue alterations due to the loss or poor organization of elastic fibers. Among these events, calcification may play a fundamental role in the rigidity of tissues and specifically in the human placenta. The studies by Zhang et al. [17] have shown that PEDF can play an important role in human placenta calcification and damage, being a determining factor in vascular diseases such as preeclampsia. The aim of this study is to observe possible changes in the expression of angiogenesis and inflammation markers as well as the changes that may occur as a result of these processes, such as the presence of calcium deposits.

## 2. Patients and Methods

### 2.1. Study Population

An observational, analytical, and prospective cohort study was conducted in which 114 women in the third trimester of pregnancy (32 weeks) were analyzed. Sixty-two were clinically diagnosed with VI. In parallel, 52 control patients without a history of VI (HC) were studied. Having signed an informed consent form, the clinical history of each woman was collected, a general physical exam was conducted, and an examination of lower extremities was conducted using echo-Doppler (Portable M-Turbo Doppler Ultrasound, SonoSite, Inc., Washington, USA) at 7.5 MHz. The VI classification in the women in the study was performed according to the Classification System for Chronic Venous Disorders (CEAP) [18]. The CEAP classification is based on clinical data that collect the broad spectrum of morphological and functional alterations of the venous system. *Inclusion criteria* were women between 18 and 39 years of age in their third trimester of pregnancy with clinical evidence of VI in the lower extremities, with a classification of C1 or higher. *Exclusion criteria* were women diagnosed with diabetes mellitus and endocrine diseases, high blood pressure, autoimmune diseases, active infectious diseases, venous malformations, heart, kidney or lung failure, preeclampsia and/or hemolysis, elevated liver enzymes and low platelets (HELLP) syndrome, intrauterine growth restriction by known causes, women with a body mass index (BMI) ≥ 25 kg/m^2^, unhealthy habits, presence of pathological lesions such as placental infarcts, avascular villi, delay in villi maturation, and chronic villitis, the appearance of any screening exclusion criteria during the previous months, and prior evidence of VI.

This study was carried out according to basic ethical principles: autonomy, beneficence, nonmaleficence, and distributive justice. The development of the study followed the standards of Good Clinical Research Practice and the principles enunciated in the last Declaration of Helsinki (2013) and the Convention of Oviedo (1997). The patients were informed of the details of the study, and each provided signed consent. The project was approved by the Clinical Research Ethics Committee of the Gómez-Ulla-UAH Defence Hospital (37/17).

### 2.2. Placental Tissue Samples

Placental tissue biopsies were obtained once the placenta was expelled. In all cases, 5 fragments of the placenta were obtained using a scalpel to ensure that the samples included multiple cotyledons. These fragments were placed into 2 different sterile tubes: one containing minimum essential medium (MEM) with 1% antibiotic/antimycotic (both from Thermo Fisher Scientific, Waltham, MA, USA) and another containing RNAlater® solution (Ambion, Austin, TX, EEUU). In the laboratory, the samples were processed in a laminar flow bench (Telstar AV 30/70 Müller class II 220 V 50 MHz; Telstar SA Group, Terrassa, Spain) in a sterile environment. The preserved samples were kept in 1 mL of RNAlater® at −80°C until processing for gene expression analysis. The samples conserved in MEM were reserved for histological and immunodetection studies.

### 2.3. Gene Expression Analysis

RNA was extracted using the guanidinium thiocyanate-phenol-chloroform method described by Ortega et al. [19]. RT-qPCR was carried out in a StepOnePlus™ System (Applied Biosystems—Life Technologies, Waltham, Massachusetts, USA) using the standard curve method. The reaction was performed as follows: 1 : 20 dilution of 5 *μ*l of each sample in nuclease-free water mixed with 10 *μ*l of DNase- and RNase-free water in a MicroAmp® 96-well plate (Applied Biosystems-Life Technologies) for a total reaction volume of 20 *μ*l. All sequences were designed *de novo* (Table 1).

### 2.4. Histological Studies

The samples that were preserved in MEM were rinsed and hydrated multiple times with antibiotic-free medium to eliminate blood cells and then were cut into fragments, which were fixed in F13 (60% ethanol, 20% methanol, 7% polyethylene glycol, 13% distilled H_2_O) according to established protocols [20]. Once included, paraffin blocks were made using molds. Once the paraffin solidified, an HM 350 S rotation microtome (Thermo Fisher Scientific, Massachusetts, USA) was used to obtain 5 *μ*m thick sections, which were spread in a hot water bath and collected on glass slides previously treated with 10% polylysine for better adhesion of the sections.

### 2.5. von Kossa Staining

von Kossa staining was utilized for the placenta samples, which allowed calcium deposits to be distinguished (seen as a brown-black color) from the remaining tissue (red color). Staining was performed according to the following protocol. The samples were stained with silver nitrate for 20 minutes, rinsed in 5% sodium hyposulfite for 15 minutes, and rinsed in running water. Then, the samples were stained with safranin for 1 minute, dehydrated in 96% alcohol for 3 minutes, dehydrated in 100% alcohol for 5 minutes, and cleared with xylol for 10 minutes. The samples were then mounted with Cytoseal™.

### 2.6. Immunohistochemical Studies

The antigen-antibody reaction was detected with the ABC method (avidin-biotin complex) with peroxidase or alkaline phosphatase as the chromogen according to the following protocol. The samples were rinsed 3 times in 1× PBS for 5 minutes each time. Nonspecific binding sites were blocked with 3% bovine serum albumin (BSA) in PBS for 30 minutes at room temperature. The samples were incubated overnight at 4°C in primary antibody diluted in 3% BSA and PBS (Table 2). Then, the samples were rinsed in PBS 3 times for 5 minutes each time. The samples were incubated in biotin-conjugated secondary antibody and diluted in PBS for 1.5 hours at room temperature (Table 3) and then rinsed in PBS 3 times for 5 minutes each time. The samples were then incubated in the avidin-peroxidase conjugate ExtrAvidin®-Peroxidase (Sigma-Aldrich, St. Louis, MO, USA) for 60 minutes at room temperature (diluted 1 : 200 in PBS) for PEDF and VEGF. For TGF-*β*1, the samples were incubated in the avidin-phosphatase conjugate ExtrAvidin®-Alkaline Phosphatase (Sigma-Aldrich, St. Louis, MO, USA) under the same conditions. The samples were rinsed in PBS 3 times for 5 minutes each time. To expose PEDF and VEGF staining, the samples were incubated in the chromogenic substrate diaminobenzidine (Kit DAB, SK-4100) (Vector Laboratories, Burlingame, CA, USA), which was prepared immediately before exposure (5 mL of distilled water, 2 drops of buffer, 4 drops of DAB, 2 drops of hydrogen peroxide), resulting in a brown stain; to expose TGF-*β*1, the samples were incubated in alkaline chromogenic substrate for 15 minutes. After exposure to chromogenic substrate, the samples were rinsed in distilled water 3 times for 5 minutes each time to stop the reaction. For contrast, nuclei were stained with Carazzi hematoxylin for 5–15 minutes. The samples were rinsed in running tap water for 10 minutes and then mounted in the aqueous polymer Plasdone. In all immunohistochemical studies, sections of the same tissue were used as a negative control, in which incubation with primary antibody was substituted with incubation in blocking solution.

### 2.7. Statistical Analysis and Interpretation of Results

For the statistical analysis, GraphPad Prism® 6.0 was used. The Mann–Whitney *U* test was applied, and the Pearson *χ*^2^ test was used. The data are expressed as the median with interquartile range (IQR). Significance was established at values of *p* < 0.05 (^*∗*^), *p* < 0.01 (^*∗∗*^), and *p* < 0.001 (^*∗∗∗*^). For each of the patients in the established groups, 5 sections and 10 fields per section were randomly selected. Patients were described as positive when the marked average area in the analyzed sample was greater than or equal to 5% of the total, according to the IRS score following the anatomical protocol of Cristóbal et al. [21]. The preparations were examined under a Zeiss Axiophot optical microscope (Carl Zeiss, Germany).

## 3. Results

### 3.1. Clinical and Demographic Characteristics

The study included 127 women with gestational VI, but 13 were excluded for not completing the study protocol or for leaving the study voluntarily. The complete study was carried out with 114 patients, including 62 women with gestational VI and 52 women without evidence of VI during pregnancy (mean age (SD) = 32.9 (3.5) VI; 34.1 (5.2) HC). No significant differences were observed between the VI and HC groups with respect to gestational age, number of previous pregnancies, previous abortions, regularity of the menstrual cycle, BMI, or size and weight of the placenta. According to the Classification System for Chronic Venous Disorders (CEAP) diagnosis [18], women with gestational VI had a score ≥ C1.

### 3.2. Expression of VEGF and TGF-*β*1

**Figure 1:**
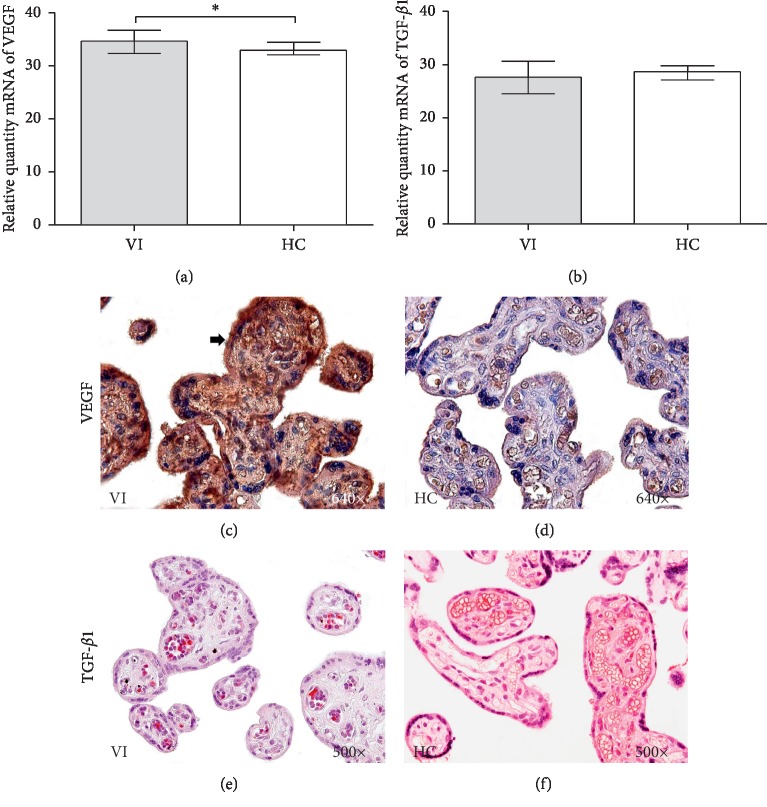
Relative quantity mRNA levels of VEGF (a) and TGF-*β*1 (b). Histological images of VEGF and TGF-*β*1 protein expression in placentas of VI (c–e) and HC (d–f). VI = lower extremity venous insufficiency; HC = control patients without VI; *p* < 0.05 (^*∗*^).

By analyzing mRNA levels with RT-qPCR, a significant increase in VEGF gene expression was observed in the placentas of women with VI compared to that in the placentas of women in the HC group (34.575 RQ (Relative quantity) [32.380–36.720] VI vs 32.965 [30.580–36.320] HC ^*∗*^*p*=0.0158) (Figure 1(a)). No significant differences were observed between the established study groups in placenta TGF-*β*1 expression (27.950 RQ [24.520–30.660] VI vs 28.665 RQ [25.870–31.480] HC *p*=0.2234) (Figure 1(b)).

The detection of VEGF protein expression by immunohistochemistry revealed high expression levels in the syncytiotrophoblast and cytotrophoblast of placental villi in women with VI (Figures 1(c) and 1(d)). A significant increase in the IRS score for VEGF was established in the placental villi of women with VI (1.500 [0.500–3.000] VI vs 1.000 [0.000–2.500] HC ^*∗*^*p*=0.0498). For TGF-*β*1, no differences were observed in terms of protein expression in the placental villi studied (0.750 [0.250–1.500] VI vs 1.000 [0.500–2.250] HC *p*=0.1497) (Figures 1(e) and 1(f)).

### 3.3. Expression of PEDF

**Figure 2:**
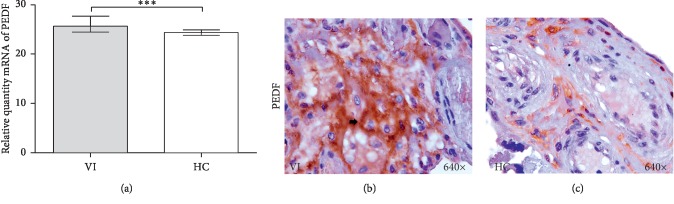
Relative quantity mRNA levels of PEDF (a). Histological images of PEDF protein expression in placentas of VI (b) and HC (c). *p* < 0.001 (^*∗∗∗*^).

PEDF gene expression was significantly higher in women with VI (25.417 RQ [24.459–27.675] VI vs 23.102 RQ [23.102–30.223] HC ^*∗∗∗*^*p*=0.0003) (Figure 2(a)). Furthermore, detection of PEDF protein expression using immunohistochemistry revealed significantly increased expression in the extracellular matrix of the high areas of the placental villi in women with VI compared to women in the HC group (Figures 2(b) and 2(c)). Analysis of the PEDF expression score showed a significant increase in protein expression (2.500 [1.000–3.000] VI vs 1.000 [0.500–3.000] HC. ^*∗∗∗*^*p* < 0.0001).

### 3.4. Study of Calcium Deposits

**Figure 3:**
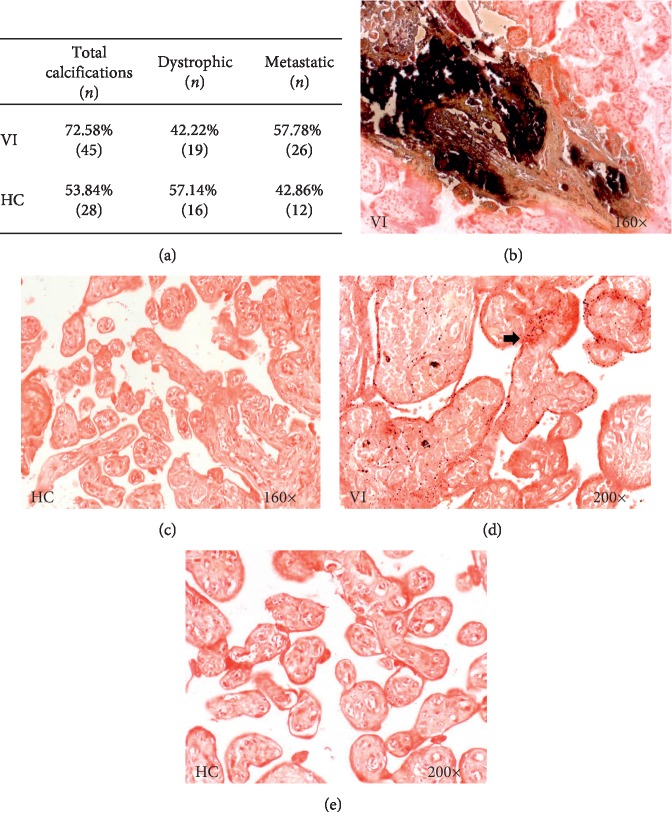
Percentage of patients with calcium deposits (a). Histological images of calcium deposits, where dystrophic (b) and metastatic (d) calcifications can be observed. VI = lower extremity venous insufficiency; HC = control patients without VI.

The study of calcium deposits in placental villi was performed using the von Kossa technique. The percentage of calcium deposits was higher in women with VI than in women in the HC group (72.58% VI vs. 53.84% HC). In this case, the Pearson *χ*^2^ test was ^*∗*^*p*=0.038 (Figure 3(a)). The histological study of calcium deposits revealed dystrophic and metastatic calcifications in the placental villi. In women with VI, the percentage of metastatic calcifications (57.78%/42.22%) was higher than that in the control group (Figures 3(b) and 3(d)). The control group presented a higher percentage of dystrophic calcifications (57.14%/42.86%) (Figures 3(c) and 3(e)).

## 4. Discussion

VI is a disorder that is difficult to approach, where the systemic and specific repercussions on maternal-fetal health are still unknown. The placenta is the tissue through which the exchange of substances essential for normal fetal homeostasis will occur; therefore, it is a dynamic organ that adapts to changes [22]. Our study is the first to demonstrate that the placentas of women with VI during pregnancy undergo changes in the expression of factors important for tissue function, such as VEGF and PEDF, and that there is a significant increase in calcification deposits in the placental villi.

Our previous studies demonstrated the existence of tissue hypoxia characterized by an increase in HIF protein and gene expression in placental villi in women with VI, which is associated with increased placental apoptosis [7]. The increase in HIF in a hypoxic condition is associated with VEGF activity [23, 24]. The increase in the activity and expression of VEGF in situations of high blood pressure, such as preeclampsia, is well known [25]. Zhang et al. [26] showed how increased VEGF activity in placentas in preeclampsia produced an increase in apoptosis. Therefore, the increase in VEGF expression in the placental villi of mothers with VI may be related to this increase in HIF activity and apoptosis.

PEDF plays an important role in vascular pathology because it is multifunctional with anti-angiogenic, anti-inflammatory, and antithrombotic properties [27, 28]. Increased expression of PEDF in the placentas of women with preeclampsia induces placental vascular reconstruction dysfunction and pathological conditions such as placental ischemia and hypoxia, which may be involved in the pathogenesis and pathogenic development of preeclampsia [16].

The gene expression levels of VEGF and PEDF coincide with those described by other authors, such as Loegl et al. for placentas in the third trimester [29]. Likewise, the tissue distribution for both proteins follows a similar pattern in our studies. The ratio of VEGF to PEDF is 3 : 2 in homeostasis. In placentas under VI, an increase of approximately 4% in the gene expression for both molecules was observed. Protein accumulation increased proportionally. However, from the point of view of tissue, the increase in PEDF in the extracellular matrix could provide the appropriate substrate for the nucleation of calcium. PEDF present in higher concentrations than usual in the extracellular matrix has been described in dermis with connective tissue alterations as especially sensitive sites for calcium nucleation [30]. In addition, in placentas of women with VI, it has been demonstrated that placental villi undergo a change in the collagen fibers in the extracellular matrix, producing an alteration in the collagen I/III ratio [9]. Therefore, all these facts seem to focus not so much on the primary role involved in the increase in VEGF/PEDF at the angiogenic level in the VI placenta but, rather, a paracrine effect related to increased calcification. Our results show that in the placentas of women with VI, the dystrophic/metastatic calcification ratio reversed compared to the placentas of women in control group, with an increase in the metastatic calcification in VI placentas.

The calcification of highly vascularized tissues undergoing hypoxic processes has been described in numerous studies, with consequences on cellular dynamics [31–33]. The process of calcification in the placenta has been described in pathological processes such as preeclampsia and intrauterine growth restriction [34, 35]. The presence of microcalcifications in placental villi seems to have an implication in events such as oxidative stress that occur in situations such as fetal anomalies and mothers with gestational hypertension, gestational diabetes, and placental abruption [36, 37]. Some authors directly relate tissue calcification with changes in cellular metabolism [38, 39]. Therefore, we speculate that the placentas of women with VI may undergo this process as a mechanism to satisfy greater cellular demand. Therefore, all these slight changes induced by possible slowing of the blood flow of the intervillous space, due to poor peripheral blood circulation in the placental environment, can manifest in tissues as increased metastatic placental calcification. This event could affect the normal metabolic exchange of the placenta in women with VI.

## Figures and Tables

**Table 1:** Sequences and binding temperatures for RT-qPCR (temp).

Gene	Sequence fwd (5′ ⟶ 3′)	Sequence rev (5′ ⟶ 3′)	Temp (°C)
GADPH	ATGACGAGGGCCTGGAGTGTG	CCTATGTGCTGGCCTTGGTGAG	60
VEGF	ATGACGAGGGCCTGGAGTGTG	CCTATGTGCTGGCCTTGGTGAG	60
TGF-*β*1	GCGTGCTAATGGTGGAAAC	CGGAGCTCTTGATGTGTTGAAGA	60
PEDF	AGTTACGAAGGCGAAGTCACCAAGTC	GCCCGGTGTTCCACCTGAGTC	50

**Table 2:** Primary antibodies that were used and their dilutions.

Antigen	Species	Dilution	Provider	Protocol specifications
VEGF	Mouse monoclonal	1 : 50	Abcam (ab28775)	—
TGF-*β*1	Rabbit polyclonal	1 : 100	Abcam (ab95866)	—
PEDF	Mouse monoclonal	1 : 500	Abcam (ab115489)	Citrate tampon in heat (pH = 6)

**Table 3:** Secondary antibodies that were used and their dilutions.

Antigen	Species	Dilution	Provider	Protocol specifications
IgG (mouse)	Goat polyclonal	1 : 300	Sigma (F2012/045K6072)	—
IgG (rabbit)	Mouse polyclonal	1 : 1000	Sigma (RG-96/B5283)	—

## Data Availability

The data used to support the findings of the present study are available from the corresponding author upon request.
